# Depression and anxiety in Hodgkin lymphoma patients: A Danish nationwide cohort study of 945 patients

**DOI:** 10.1002/cam4.2981

**Published:** 2020-04-17

**Authors:** Andreas K. Øvlisen, Lasse H. Jakobsen, Kristian H. Kragholm, René E. Nielsen, Martin Hutchings, Rasmus B. Dahl‐Sørensen, Henrik Frederiksen, Danny Stoltenberg, Martin Bøgsted, Lene S. G. Østgård, Marianne T. Severinsen, Tarec C. El‐Galaly

**Affiliations:** ^1^ Department of Haematology Aalborg University Hospital Aalborg Denmark; ^2^ Department of Clinical Medicine Aalborg University Aalborg Denmark; ^3^ Department of Cardiology Unit of Epidemiology and Biostatistics Aalborg University Hospital Aalborg Denmark; ^4^ Department of Psychiatry Aalborg University Hospital Aalborg Denmark; ^5^ Department of Haematology Rigshospitalet Copenhagen University Hospital Copenhagen Denmark; ^6^ Department of Haematology Zealand University Hospital Roskilde Denmark; ^7^ Department of Haematology Odense University Hospital Odense Denmark; ^8^ Department of Haematology Copenhagen University Hospital Herlev Denmark; ^9^ Department of Haematology Aarhus University Hospital Aarhus Denmark; ^10^ Clinical Cancer Research Unit Aalborg University Hospital Aalborg Denmark

**Keywords:** anxiety, depression, epidemiology, Hodgkin lymphoma, psychotropic drugs

## Abstract

Cancer‐related psychological distress may lead to depression and anxiety among survivors. The vast majority of patients with Hodgkin lymphoma (HL) become long‐term survivors, but the risk of mental health problems after HL is not well‐characterized. Using national population‐based registries, we investigated the cumulative incidence of psychotropic drug (antidepressants, antipsychotics, and anxiolytics) use (proxies for depression and anxiety) in HL patients as well as if an increased risk would normalize over time for patients in remission. The study included 945 HL patients aged 18‐92 years and 4725 matched persons. In total, 215 HL patients (22.8%) received a prescription of any psychotropic drug (PD) at some point after date of diagnosis compared to 545 persons (11.5%) in the matched cohort. Cumulative incidences with death/relapse as competing risk confirmed that HL patients were at higher risk of receiving psychotropic drug prescriptions, but the increased risk was transient and normalized to the matched population 5 years into survivorship. Increased age, Eastern Cooperative Oncology Group performance status, and disease stage were associated with higher risk of psychotropic drug prescriptions. Given the increased rate of psychotropic drug prescriptions after HL diagnosis, screening for symptoms of depression and anxiety is warranted after HL diagnosis and first years into survivorship.

## Graphical abstract

In this Danish nationwide cohort study of 945 Hodgkin lymphoma patients, we investigated the risk of anxiety and depression following diagnosis, using psychotropic drug prescriptions as proxy for anxiety and depression. Our findings showed that Hodgkin lymphoma patients had higher 5‐year cumulative incidence of receiving a prescription for a psychotropic drug (21.5%) as compared to a matched background population (8.4%).

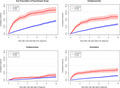

## 1 INTRODUCTION

Cancer‐related psychological distress is well described, and several studies have consistently found increased risk of depression and anxiety among cancer patients as compared to persons without cancer, which can lead to increased use of psychotropic drugs (PDs) in patients with cancer.^1^, ^2^, ^3^, ^4^, ^5^, ^6^, ^7^, ^8^, ^9^ Poor mental health, and, in particular, unrecognized depression and anxiety could substantially reduce quality of life in patients surviving cancer and might even increase mortality.^10^, ^11^ Depression and anxiety may also have detrimental impact on socioeconomic outcomes due to prolonged sick leave, increased risk of disability pension, and difficulties in maintaining family life.^12^, ^13^, ^14^, ^15^ Thus, attention to the mental health problems is important to ensure good quality of life following a cancer diagnosis.

In general, female sex and higher age are both associated with higher lifetime risk of depression and anxiety in the general population.^5^, ^6^, ^16^, ^17^ However, in patients surviving cancer, the risk of depression and anxiety is dependent on the type of cancer, that is, incidence of depression and anxiety is higher in patients with gynecological or lung cancer compared to patients with prostate or skin cancer, possibly reflecting the more favorable outcomes of the latter two.^6^, ^17^ Research focusing in particular on occurrence of depression and anxiety following hematological cancers is limited.^6^, ^18^, ^19^, ^20^, ^21^

Hodgkin lymphoma (HL) is a rare, B cell‐derived malignancy characterized by an incidence peak in younger adults of age 18‐30 years and a second incidence peak after the age of 50 years.^22^, ^23^, ^24^ Contemporary combined modality treatments of HL result in a 5‐year survival rate of 87.6%.^24^, ^25^ Universally high survival rates, regardless of disease stage, in patients tolerating multiagent chemotherapy regimens warrant research in HL survivorship with delineation of potential health issues faced by patients. To study the risk of mental health problems after diagnosis of HL, we compared the cumulative incidence of depression and anxiety among the Danish patients with HL and the matched population using prescription of PDs (antidepressants, anxiolytics, and antipsychotics) as proxies for depression and anxiety. Secondly, we investigated the patterns of PD prescriptions by time elapsed since diagnosis to analyze if a potential initial increase in PD prescriptions after diagnosis would return to rates as found in the matched population.

## 2 MATERIALS AND METHODS

### 2.1 Data sources

All Danish residents are assigned a unique personal 10‐digit Civil Personal Register (CPR) number at birth or immigration, which enables linkage of various population‐based registries. The Danish Civil Registration System includes demographic information such as age, sex, marital status, citizenship, and municipality of residence since 1960.^26^, ^27^, ^28^ The Danish Education Register, established in 1910, holds information on educational level of Danish citizen.^29^ The Danish National Patient Registry includes data for all inpatient‐, outpatient‐, and emergency department contacts in Danish hospitals since 1977 and uses ICD‐10 for coding diagnoses.^30^ The National Prescription Registry holds data on all claimed prescription from Danish pharmacies since 1994 (complete since 1995). The registry uses the global Anatomical Therapeutic Chemical classification (ATC) code for identifying drugs.^31^, ^32^ In this study, any prescription of antidepressants (ATC ‐ N06A), antipsychotics (ATC ‐ N05A), and anxiolytics (ATC ‐ N05B) were used as proxies for depression and anxiety. The Danish Lymphoma Registry (LYFO) is a nationwide registry containing information on lymphoma patients diagnosed and treated at departments of hematology in Denmark. The coverage of LYFO is 94.9%. Data include detailed disease‐related information (lymphoma subtype, stage, risk category, sites of involvement, etc), treatment information, and outcomes. Data completeness and accuracy are high with completeness ranging between 92% and 100% and positive predictive values ranging between 87% and 100%.^33^

### 2.2 Study population

A cohort of patients with HL diagnosed with both classical and lymphocyte‐predominant HL between 01 January 2005 and 31 December 2015 aged ≥18 years was identified using LYFO. Patients with fictive CPR number (foreign citizens) and patients diagnosed prior to immigration to Denmark were excluded as follow‐up was not available. Date of HL diagnosis was set as inclusion date, in the following referred to as the index date. Patients who received a prescription of PDs within 10 years prior to the index date were excluded in order to describe the incident use of PDs. For each patient with HL, five random persons from the Danish population were matched on year and month of birth and sex and included in a control cohort (background population). Inclusion date for the matched persons was the index date of the index patient. Furthermore, matched persons had to be alive and living in Denmark at the index date with no prescriptions of PDs within 10 years prior to that date. Matched persons later being diagnosed with HL were censored at the time of HL diagnosis. All included in the final study population where followed until event (first PD prescription), death, or censored at the end of follow‐up, emigration out of Denmark, or reported missing.

### 2.3 Statistical analysis

Baseline characteristics at time of inclusion were described by proportions for categorical variables, while continuous variables were described by medians with interquartile ranges, summarized separately for patients and the background population. Difference between baseline characteristics in HL patients as compared to the matched background population where described using Pearson's Chi‐square test, Fischer's exact test, and Mann‐Whitney *U* test. Time to first PD prescription was computed for patients and the matched population and presented using cumulated incidence curves. Furthermore, 5‐year cumulative incidences were computed using the Aalen‐Johansen estimator with deaths, relapses, or a matched person being diagnosed with HL before PD prescription treated as competing risk, and presented by a forest plot (with 95% confidence intervals (CIs)) for HL patients stratified on gender, age, Ann Arbor stage, Eastern Cooperative Oncology Group (ECOG) performance status, Charlson Comorbidity Index (CCI), treatment regimen, and educational level. Significant difference between the patient's and the matched population's cumulative incidence was tested using Gray's test.^34^ The pseudo‐observation method was used to compute 5‐year cumulative incidences at various time points after diagnosis to estimate differences in PD prescriptions over time.^35^, ^36^ In order to do so, all patients with HL alive at 1‐, 2‐, and 5 years after the index date without any PD prescriptions were rematched to five random persons from the matched population using the same procedure as previously described. Pseudo‐observations for cumulative incidences regarding both HL patients and the matched cohort were computed. Association between groups and outcomes (PD prescriptions) was further evaluated using an adjusted Cox proportional hazards regression analysis. Variables for adjustment were chosen before analysis according to clinical relevance and known prognostic importance including age, sex, and educational level. Hazard ratios (HRs) were calculated. Throughout the study, 95% CIs were reported and used as level of statistically significant.

Statistical analyses were conducted using SAS version 9.4 (SAS Institute Inc) and RStudio version 1.1.447 (RStudio, Inc) and R version 3.6.1 (R foundation for Statistical Computing).

All analysis was performed on pseudo‐anonymized data using the secured network governed by Statistics Denmark. The study was approved by the Danish Data Protection Agency.

## 3 RESULTS

### 3.1 Baseline characteristics

We identified 945 patients with HL who fulfilled the inclusion criteria. Median age was 39 years and the male:female ratio was 1.7. Median follow‐up was 7.2 (7.0‐7.4) years for HL patients (reverse Kaplan‐Meier method). Included patients with HL were matched to 4725 persons from the background population Table 1 shows baseline information for both patients with HL and the background population including age, disease stage, CCI score, and ECOG performance status. A statistically significant difference (*P*‐value <.05) between HL patients and the background population was found for CCI score, educational level, and PD use (both overall and stratified on PD type).

**TABLE 1:** Baseline characteristics and psychotropic drug use in patients with HL and the background population

	Patients with HL (n = 945)	Background population (n = 4725)	*P*‐value
Age, n (%)			
Median (IQR)	39 (27‐59)	39 (27‐59)	1.000
18‐ to 30‐year‐old	288 (30.5%)	1440 (30.5%)	1.000
31‐ to 60‐year‐old	407 (43.1%)	2035 (43.1%)	
61‐year‐old	250 (26.5%)	1250 (26.5%)	
Sex, n (%)			
Male	591 (62.5%)	2955 (62.5%)	1.000
Female	354 (37.5%)	1770 (37.5%)	
CCI score prior to diagnosis, n (%)			
0	771 (81.6%)	4189 (88.7%)	<.001*
≥1	174 (18.4%)	536 (11.3%)	
Ann Arbor stage, n (%)			
Limited stage (I‐II)	517 (54.7%)	NA	NA
Advanced stage (III‐IV)	424 (44.9%)	NA	
ECOG performance status, n (%)			
0	670 (70.9%)	NA	NA
1‐4	271 (28.7%)	NA	
Treatment			
2‐4 cycles ABVD	312 (33.0%)	NA	NA
6‐8 cycles ABVD	375 (39.7%)	NA	
6‐8 cycles BEACOPP	94 (9.9%)	NA	
Other	91 (9.6%)	NA	
Missing	73 (7.7%)	NA	
Educational level (ISCED), n (%)			
Primary education	399 (42.2%)	1597 (33.8%)	<.001*
Secondary/tertiary education	500 (52.9%)	2210 (46.8%)	
Missing	46 (4.9%)	918 (19.4%)	
Any psychotropic drug prescription overall, n (%)			
Yes	215 (22.8%)	545 (11.5%)	<.001*
No	730 (77.2%)	4180 (88.5%)	
Antidepressant prescription, n (%)			
Yes	154 (16.3%)	404 (8.6%)	<.001*
No	791 (83.7%)	4321 (91.4%)	
Antipsychotic prescription, n (%)			
Yes	37 (3.9%)	129 (2.7%)	.011*
No	908 (96.1%)	4596 (97.3%)	
Anxiolytic prescription, n (%)			
Yes	93 (9.8%)	189 (4.0%)	<.001*
No	852 (90.2%)	4536 (96.0%)	
Days between first and last psychotropic drug prescription, median (IQR)	113 (0‐888)	208 (0‐1018)	.358
Days between first and last antidepressant prescription, median (IQR)	157.5 (0‐701)	312.5 (1.5‐1055.75)	.204
Days between first and last antipsychotic prescription, median (IQR)	0 (0‐255)	50 (0‐672)	.069
Days between first and last anxiolytic prescription, median (IQR)	0 (0‐330)	0 (0‐99)	.141

Abbreviations: ABVD, doxorubicin, bleomycin, vinblastine, and dacarbazine; BEACOPP, bleomycin, etoposide, doxorubicin, vincristine, procarbazine, and prednisone; CCI, Charlson Comorbidity Index; ECOG, Eastern Cooperative Oncology Group; HL, Hodgkin lymphoma; IQR, interquartile range; ISCED, International Standard Classification of Education.

* Statistical significant (*P*‐value <.05).

### 3.2 Incident PD use

In total, 215 (22.8%) of the patients with HL received at least one prescription for any PD compared to 384 (11.5%) within the background population (Table 1). Antidepressants were the most frequent prescribed PD type (16.3% for patients with HL and 8.6% for background population). If including hypnotics and sedatives (ATC – N05C) as anxiolytics (due to the benzodiazepine‐like nature of hypnotics and sedatives), anxiolytics would be the far most prescribed PD in patients with HL (23.9% compared to 10.0% in the background population; data not presented).

**FIGURE 1:**
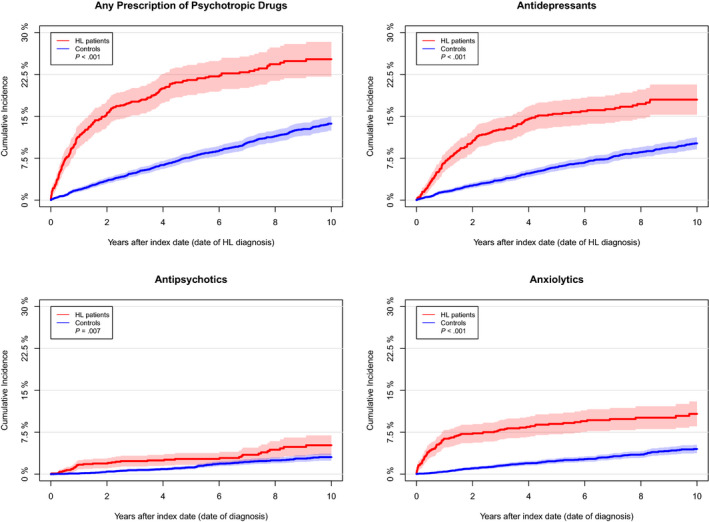
Cumulative incidence curves of time to first prescription of psychotropic drugs (PDs) (antidepressants, antipsychotics, anxiolytics) and time to the first of any PD for all patients stratified on patients with HL and the matched cohort. Gray's test showed significant difference between cumulated incidences for HL patients and the background population regarding all four cumulated incidence curves (described in figure legends)

Figure 1 shows the cumulative incidences of time to first PD prescription stratified by type of PD. Patients with HL had higher cumulative incidence of PD prescriptions compared to the background population, which was also illustrated in a crude Cox regression analysis (HR 2.63; CI 2.24‐3.08; *P* < .001; Table 2).

**TABLE 2:** Association between use of psychotropic drugs in HL and clinical variables

	HR	95% CI	*P*‐value
Patients with HL vs background population			
Controls	1.00	(reference)	
Cases	2.63	2.24‐3.08	<.001*
Sex^a^			
Male	1.00	(reference)	
Female	1.21	0.93‐1.59	.163
Age‐group^a^			
18‐ to 30‐year‐old	1.00	(reference)	
31‐ to 60‐year‐old	1.69	1.19‐2.40	.004*
≥61‐year‐old	2.57	1.77‐3.75	.007*
ECOG performance status^a^			
0	1.00	(reference)	
≥1	2.19	1.66‐2.89	<.001*
Ann Arbor Stage^a^			
Limited stage (I‐II)	1.00	(reference)	
Advanced stage (III‐IV)	1.84	1.41‐2.41	<.001*
CCI‐score^a^			
0	1.00	(reference)	
≥1	1.53	1.10‐2.12	.011*
Treatment^a^			
2‐4 cycles ABVD	1.00	(reference)	
6‐8 cycles ABVD	1.45	1.05‐2.00	.024*
6‐8 cycles BEACOPP	0.95	0.56‐1.61	.857
Other	1.83	1.15‐2.92	.011*
Missing	1.49	1.15‐2.92	.166
Educational level (ISCED)^a^			
Primary education	1.00	(reference)	
Secondary/tertiary education	1.11	0.84‐1.46	.466

Abbreviations: ABVD, doxorubicin, bleomycin, vinblastine, and dacarbazine; BEACOPP, bleomycin, etoposide, doxorubicin, vincristine, procarbazine, and prednisone; CCI, Charlson Comorbidity Index; CI, confidence interval; ECOG, Eastern Cooperative Oncology Group; HL, Hodgkin lymphoma; HR, hazard ratio; IQR, interquartile range; ISCED, International standard classification of education.

^a^ Only HL patients are included in Cox regression analysis.

* Statistically significant (*P*‐value <.05).

A sensitivity analysis was performed, in which incident PD use was defined as having received at least two prescriptions of PDs. The 5‐year cumulative incidence is presented in Figure S1. Results of the sensitivity analysis are consistent with the primary analysis results.

An additional analysis, in which the cumulative incidence of time to first PD prescription from 5 years prior to index date till 5 years after the index date was performed to investigate from which time point the use of PDs among HL patients departed from the use in the matched background population. This analysis did not exclude HL patients with PD prescriptions prior to HL diagnosis. The cumulative incidence (Figure S2) among HL patients gradually departed from the use of PDs in the background population already 2 years prior to index date and strongly increased in the year prior to diagnosis.

### 3.3 Incident PD use in relapsed HL patients

In total, 117 HL patients (12.4%) experienced relapse during follow‐up. To address the use of PDs in relapsed HL patients, patients with relapsed HL and without PD use 6 months prior to the relapse date were rematched to the background population with new index date being the date of relapse. The 5‐year cumulative incidence with death without PD prescription as competing risk is presented in Figure S3, which shows an overall higher use of PDs in relapsed HL patients at 5 years after relapse date (27.1%) compared to the background population (7.7%) as well as a higher use of antidepressants (18.8% vs 5.7%), antipsychotics (6.0% vs 1.5%), and anxiolytics (12.8% vs 2.4%).

### 3.4 Association between PD, patients’ characteristics, and clinicopathologic features

**FIGURE 2:**
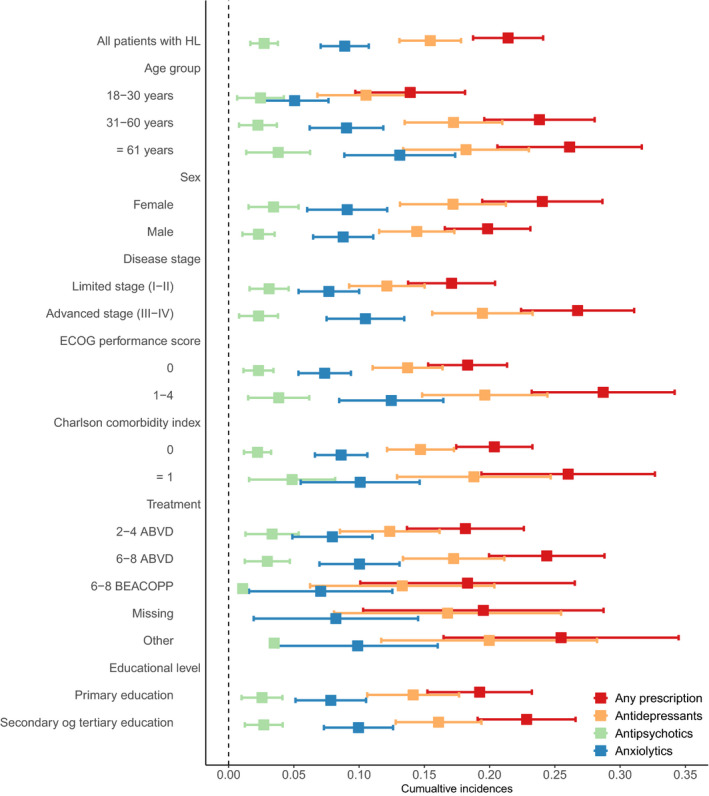
Forest plot showing 5‐year cumulative incidences provided with 95% confidence intervals of use of psychotropic drugs (PDs) for clinical variables in patients with Hodgkin lymphoma (HL) stratified on type of PD

The 5‐year cumulative incidences of PD prescriptions stratified on the type of PD in the matched cohort (Figure S4) showed that age >60 years, females, and CCI score ≥1 all were associated with higher rates of PD prescriptions. Educational level did not show any difference in the background population. Figure 2 shows stratified 5‐year cumulative incidences among HL patients according to age, sex, disease stage, ECOG performance status, CCI, and education. Age >30 years, advanced stage disease, and ECOG performance status >1 at diagnosis were all associated with higher rates of PD prescriptions. The associations were mainly driven by the use of antidepressants.

Crude Cox regression analysis for the association between clinical features and PD prescriptions among HL patients is presented in Table 2. Age (HR 1.69; CI 1.19‐2.40 in 31‐ to 60‐year‐old and HR 2.57; CI 1.77‐3.75 in ≥61‐year‐old with age 18‐30 years as reference), ECOG performance status ≥1 (HR 2.19; CI 1.66‐2.89), advanced stage disease (HR 1.84; CI 1.41‐2.41), CCI score ≥1 (HR 1.53, CI 1.10‐2.12), and treatment with 6‐8 cycles of ABVD (doxorubicin, bleomycin, vincristine, and dacarbazine) (HR, 1.45; CI 1.05‐2.00) were all significantly associated with PD prescriptions, whereas sex and educational level were not associated with the use of PDs.

### 3.5 Time to normalization of PD prescriptions

**FIGURE 3:**
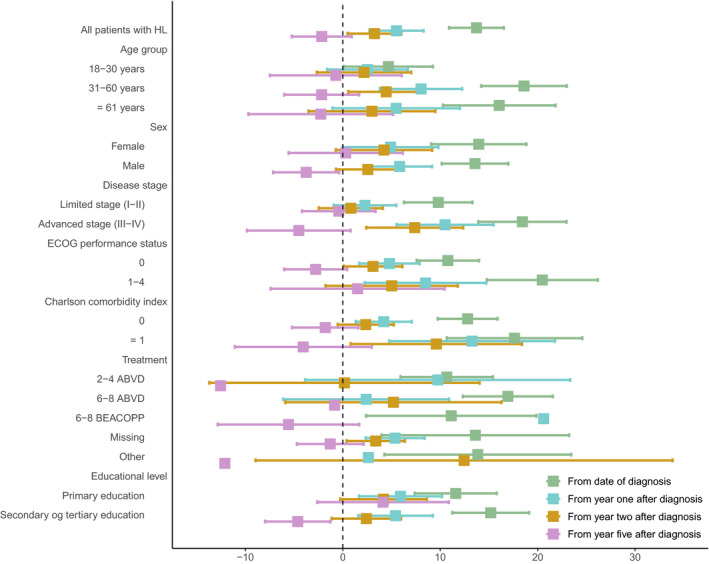
Five‐year excess cumulative incidences of psychotropic drug (PD) use from index date and later time points for all patients with HL and by subgroups of patients compared to an age‐sex matched cohort from the background population

Differences in cumulative incidences of PD prescriptions between HL patients and the background population computed from 1‐, 2‐, and 5 years after the index date are presented in Figure 3. The differences gradually diminished as time elapsed and 5 years after diagnosis, patients with HL had similar rate of incident PD prescriptions as the background population.

## 4 DISCUSSION

This nationwide study demonstrates that 5‐year cumulative incidence of PD prescriptions was increased among patients with HL (21.5%) compared to the background population (8.4%). These data support results from other studies showing higher risk of depression and anxiety in patients with cancer.^1^, ^4^, ^5^, ^6^, ^7^, ^8^, ^9^ Risk factors for PD prescription were increasing age, advanced disease stage, CCI score ≥1, poor ECOG performance status (≥1), and patients treated with 6‐8 cycles of ABVD. However, treatments differ in length, why interpretation of the results must be careful. Importantly, the increased risk of mental health problems leading to PD prescriptions appears to be transient as cumulative incidence of PD prescriptions among HL patients surviving the initial 5 years after diagnosis without receiving PD prescriptions becomes similar to that of a matched background population. Most interestingly, Figure S2 showed that the cumulative incidence of first PD prescription was higher for HL patients already from 2 years prior to index date. To our knowledge, PDs have not been shown as a risk for developing HL. Furthermore, Figure S2 showed a rapidly increasing cumulative incidence during the months prior to the index date. The reason may be that insidious HL symptoms, such as fatigue, weight loss, and night sweats (B‐symptoms), could initially be misinterpreted as mental health problems such as stress or depression. Additionally, the present study shows that HL patients with relapse have markedly higher 5‐year cumulative incidence (27.1%) than that of the background population (7.7%), as well as the overall population of HL population (22.8%), which might be explained by the more stressful situation with higher degree of uncertainty faced by relapsed HL patients.

In a previous study by Linden et al,^6^ a routine evaluation of mental health was carried out at British Columbia Cancer Agency centers by distributing questionnaires to patients with various types of cancers prior to treatment. In total, 19% of 9394 patients had clinical anxiety according to the Psychosocial Screen for Cancer (PSSCAN),^37^ while 12.9% patients had clinical depression. In the subgroup with hematological cancers, the prevalence of anxiety was 38/167 (22.8%) and the prevalence of depression was 28/166 (16.9%). Our results resemble those by Linden et al,^6^ although the authors did not provide detailed data on mental health problems according to type of hematological cancer. As hematological cancers are highly diverse in terms of age at diagnosis, symptomatic burden, treatments, and prognosis, some differences in risk of mental health problems would be expected.

Conte et al^2^ investigated the incident use of PDs in all subtypes of non‐Hodgkin B‐cell lymphoma patients (B‐NHL) from date of diagnosis and 8 months onward in France. In total, 745 incident B‐NHL patients with a mean age of 65.1 years were included. In this study, 31.5% of the patients had at least one prescription for a PD, after a median time from diagnosis of approximately 2 months, and younger age was associated with higher PD use. The proportion of patients receiving PDs was markedly higher than in our study. Our results regarding age are reciprocal to the results of Conte et al,^2^ as higher age in patients with HL was associated with higher cumulative incidence of PD prescriptions. The difference might be caused by the significant difference in prognostic outcomes between B‐NHL and HL patients. B‐NHL has lower expected 5‐year survival combined with high risk of relapse as compared to HL across all ages. Hereby, older B‐NHL patients are more likely to die before having the opportunity for receiving a PD prescription. Also, as young B‐NHL patients have both lower cure rates and higher risk of relapse as compared to young HL patients, the risk of affected mental health may be higher in B‐NHL patients given the worse prognostic outcomes.

The psychological health measured by fatigue level, physical functioning, social functioning, cognitive problems, and quality of life has been assessed in HL survivors in other studies.^38^, ^39^, ^41^, ^42^ Hjermstad et al^38^ showed a higher prevalence of chronic fatigue in HL survivors compared to a Norwegian normative population (30% vs 11%). These findings have been confirmed by Loge & Kaasa^20^ and Daniëls et al^18^ in which Daniëls et al^18^ found an association between chronic fatigue and depression/anxiety in HL patients. Furthermore, systematic reviews by [39, 41, 42] investigated the impact of health‐related quality of life in HL survivors and cancer‐related fatigue. The authors found that HL survivors were more likely to report problems with physical, social, and cognitive functioning, general health, and fatigue, whereas problems directly related to mental health was not different from the background population. As more self‐reported general and psychological health problems have been shown among HL patients along with an increased incidence of depression and anxiety, a few studies looked into the risk of committing suicide in HL patients relative to the European general population.^43^, ^44^ Both studies showed a higher suicidal risk in HL patients, and ^43^ identified male HL patients as having higher risk of suicide than female HL patients. However, suicide is a rare cause of death in Denmark, why analysis on this matter could not be performed (total number of suicides for both HL patients and the matched background population ≤3).

A strength of this study is the use of high‐quality nationwide population‐based registries characterized by high coverage ensuring all HL patients eligible for inclusion were analyzed. This minimizes the risk of selection bias and ensures near to complete follow‐up of all patients. Among important limitations of the study are the reliance on PD prescription data as proxies for depression and anxiety. National guidelines for treatment of depression states that pharmacological treatment should only be initiated if severity of depression is moderate or severe, while psychotherapy or psychoeducation should be initiated at all grades of depression severity.^45^ As we used PD prescriptions as proxies for depression and anxiety, we would only include patients with moderate or severe depressions according to the National guidelines regarding pharmacological treatment of depression. However, this bias exists in both the patient cohort as well as the background population and may have less impact on estimate differences in cumulative incidence rates. On contrary, as HL patients would be seen by doctors more often than the average Danish citizen during both the treatment and the follow‐up program, surveillance bias may be introduced. Secondly, we did not account for the amount of prescriptions received. Hereby, patients only receiving one prescription of, that is, antidepressants would be defined as having depression, even though one prescription would not be enough for treating depression of any severity. However, a sensitivity analysis defining PD use as having had at least two PD prescriptions did not alter the conclusion. Instead of clinical depression, patients might suffer from adjustment disorders which have symptoms similar to major depression disorder. Hence, HL patients may have received PD prescriptions due to adjustment disorder and overt depression. This could be supported by the fact that the median number of days between the first and last prescription of antidepressants was shorter among HL patients (113 days, IQR 0‐888, Table 1) compared to the matched cohort (208 days, IQR 1.5‐1018, Table 1). This is supported in a meta‐analysis showing that adjustment disorders are highly prevalent in both oncological and hematological care settings with a prevalence of 19.4% during the first 5 years after diagnosis.^7^ Thirdly, the Danish Prescription Registry does not contain information regarding the clinical indications for the prescriptions. As PDs are widely used for other purposes, that is analgesics in neuropathic pain management, this might result in an overestimation of depression and anxiety, although studies indicate a low incidence of both neuropathy and neuropathic pain.^47^, ^48^, ^49^

In conclusion, the present study showed a higher cumulative incidence of PD prescriptions among HL patients, which likely indicates a higher incidence of mental health problems possibly due to depression and anxiety triggered by the stressful situation. Therefore, focus on the mental health in HL patients is important from a clinically perspective, but according to the findings of this study, this is relevant mainly during the initial years after diagnosis where risk is significantly higher than that of the background population. To firmly address the specifics of mental health problems in HL patients, prospective studies using validated screening procedures for psychiatric disorders in cancer patients (ie, Major Depression Inventory (MDI) questionnaire, Patient Health Questionnaire (PHQ), Hospital Anxiety and Depression Scale (HADS), and Generalized Anxiety Disorder (GAD‐2) scale^50^, ^51^) are warranted and the effects of different interventions, including pharmacological treatment and psychological support, need to be established to provide HL patients with the best possible support.

## CONFLICT OF INTEREST

AKØ travel expenses from Pfizer and AbbVie. TEG was employed by Roche, Basel from 1 January 2019. The present work was done independent of this employment and was in relation to affiliation to Aalborg University as clinical professor in hematology. REN has received research grants from H. Lundbeck and Otsuka Pharmaceuticals for clinical trials, received speaking fees from Bristol‐Myers Squibb, Astra Zeneca, Janssen & Cilag, Lundbeck, Servier, Otsuka Pharmaceuticals, Teva A/S, and Eli Lilly, and has acted as advisor to Astra Zeneca, Eli Lilly, Lundbeck, Otsuka Pharmaceuticals, Takeda, and Medivir.

## ETHICAL STATEMENT

All analysis was performed on pseudo‐anonymized data using the secured network governed by Statistics Denmark. The study was approved by the Danish Data Protection Agency (ID‐number 2018‐88).

## Supporting information

Figure S1

Figure S2

Figure S3

Figure S4

## Data Availability

Research data are not shared.
