# Supplementary figures and images for: Depression and anxiety in Hodgkin lymphoma patients: A Danish nationwide cohort study of 945 patients
Source: Cancer Med. 2020 Apr 17;9(12):4395–404. doi: 10.1002/cam4.2981 (PMC7300408; doi:10.1002/cam4.2981)

**Any Prescription of Psychotropic Drugs**

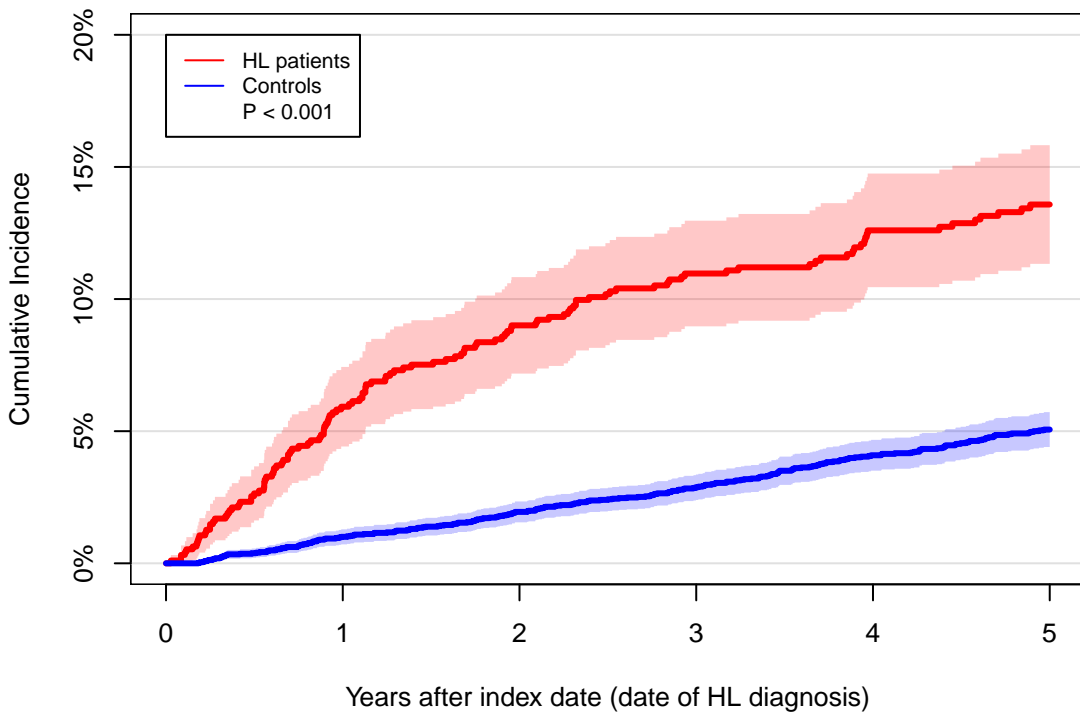

**Antidepressants**

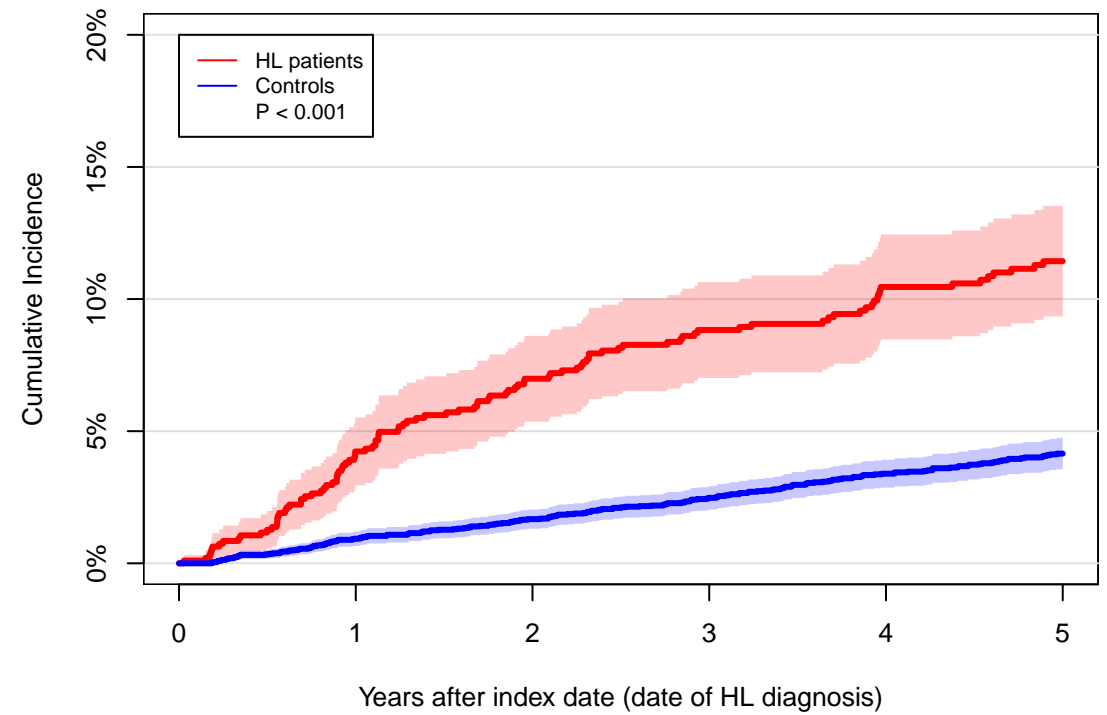

**Antipsychotics**

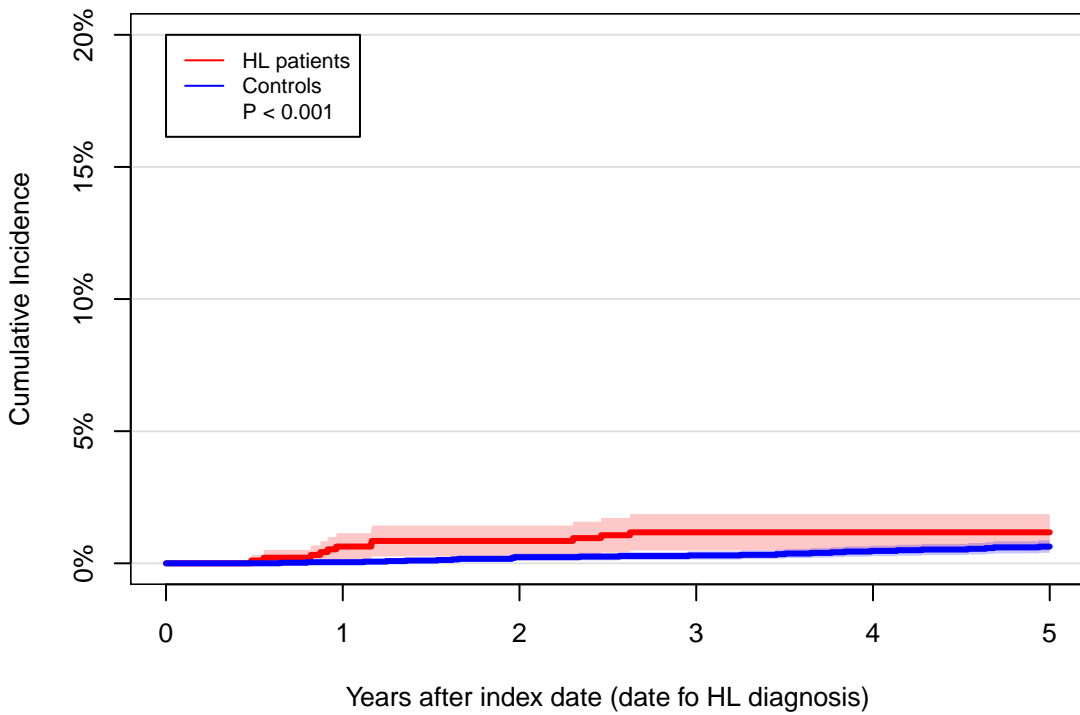

**Anxiolytics**

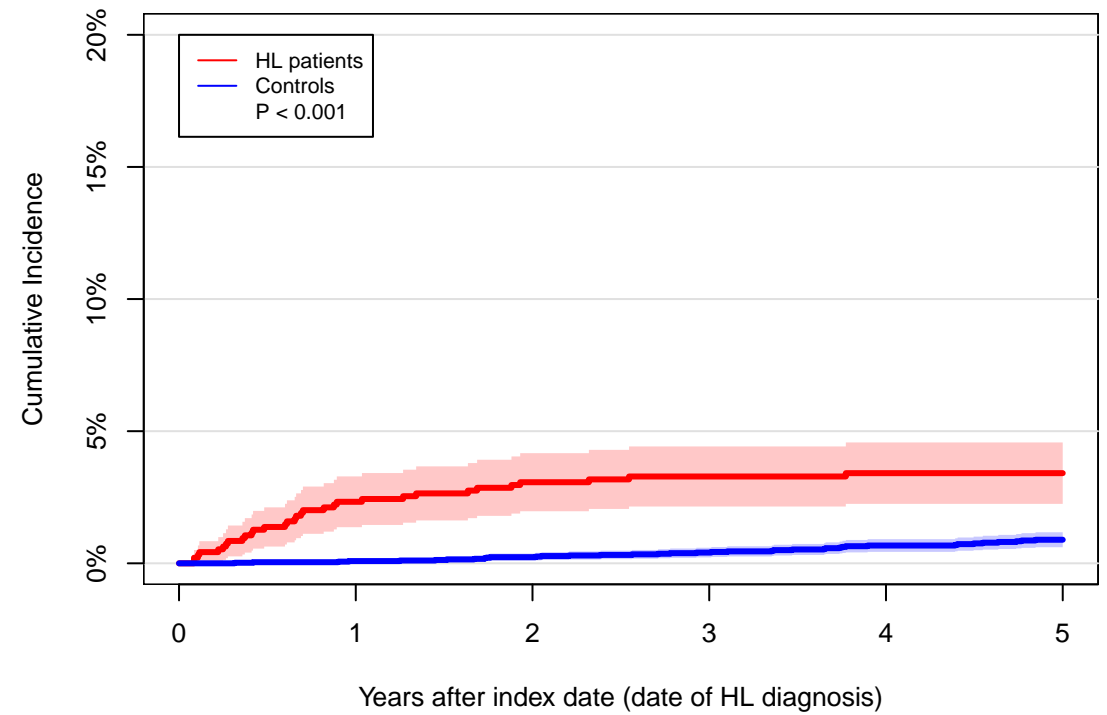

Supplement: Supplementary file 1 — Figure S1 [file CAM4-9-4395-s001.pdf]

## Any prescription of Psychotropic Drugs

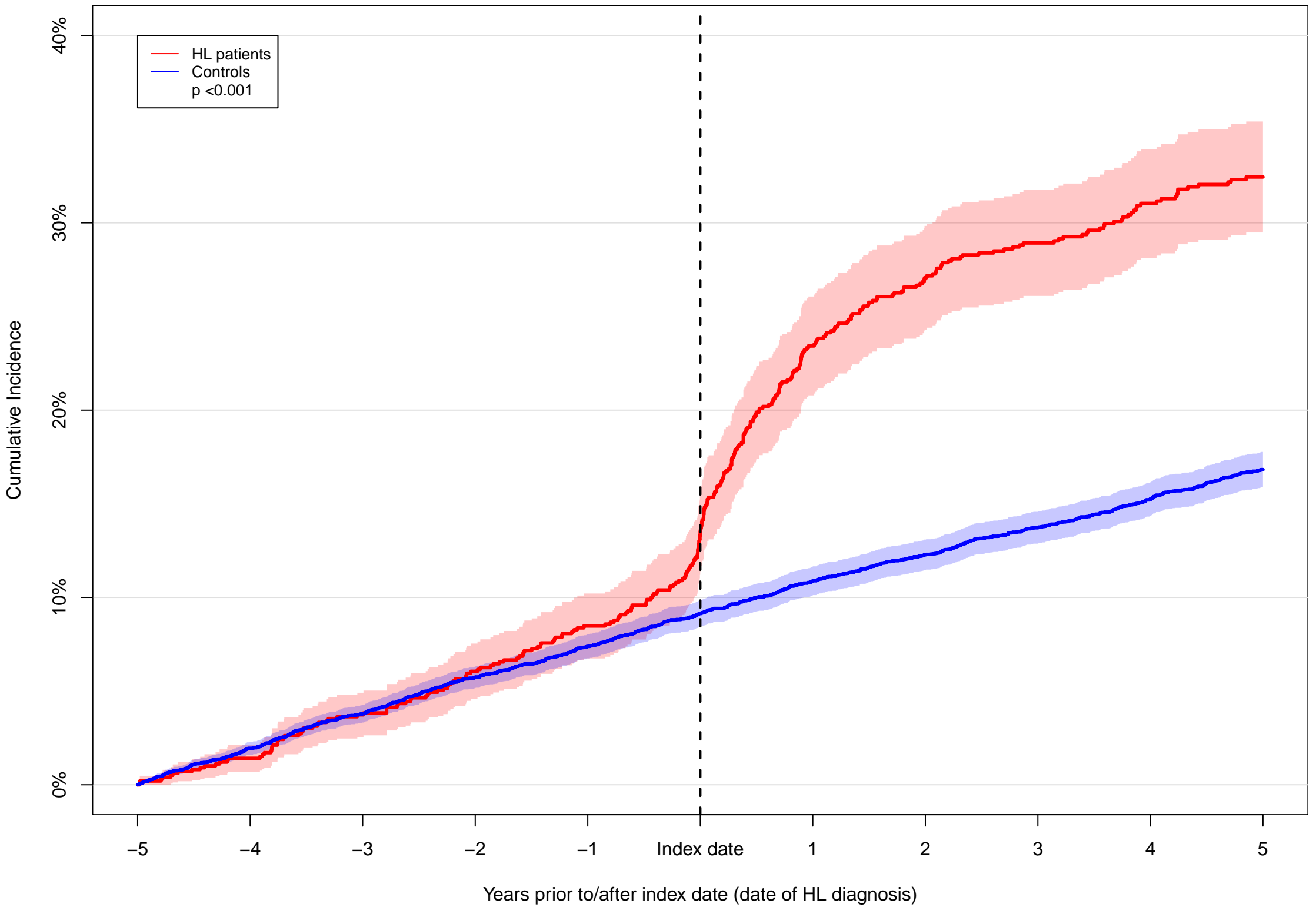

Supplement: Supplementary file 2 — Figure S2 [file CAM4-9-4395-s002.pdf]

**Any Prescription of Psychotropic Drugs**

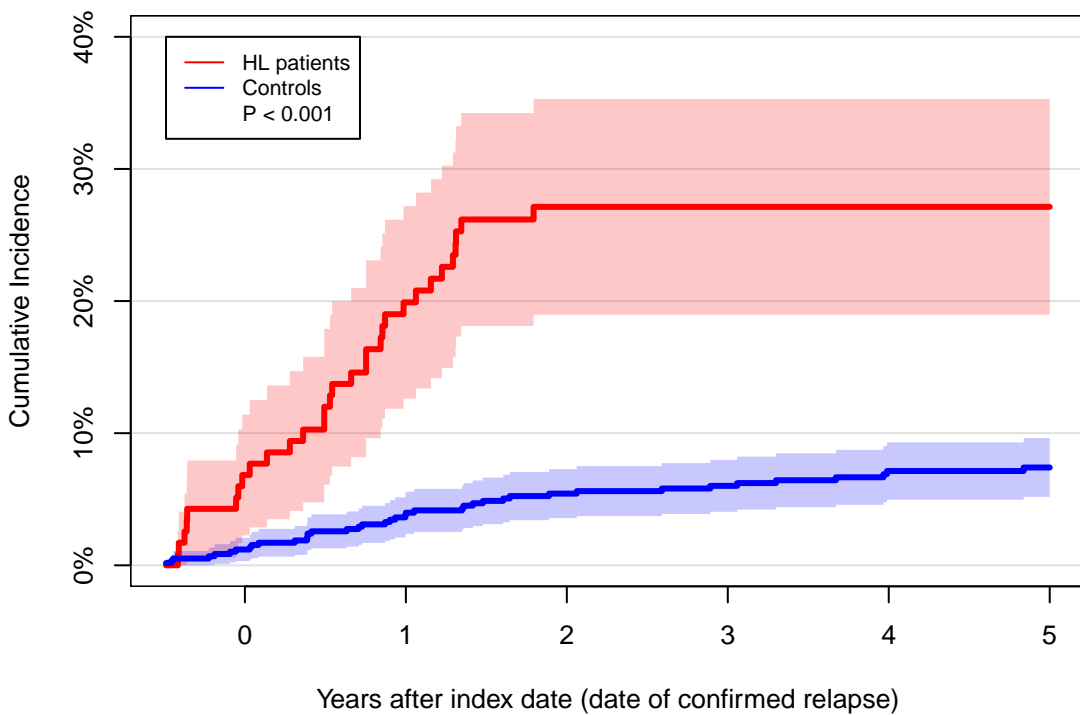

**Antidepressants**

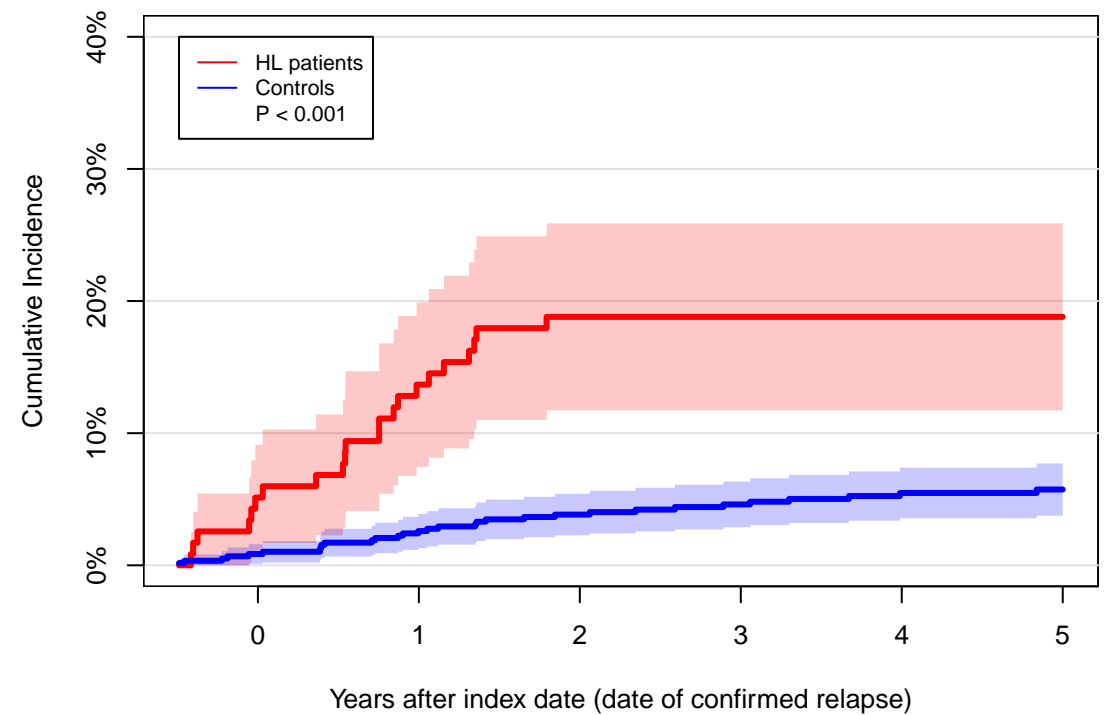

**Antipsychotics**

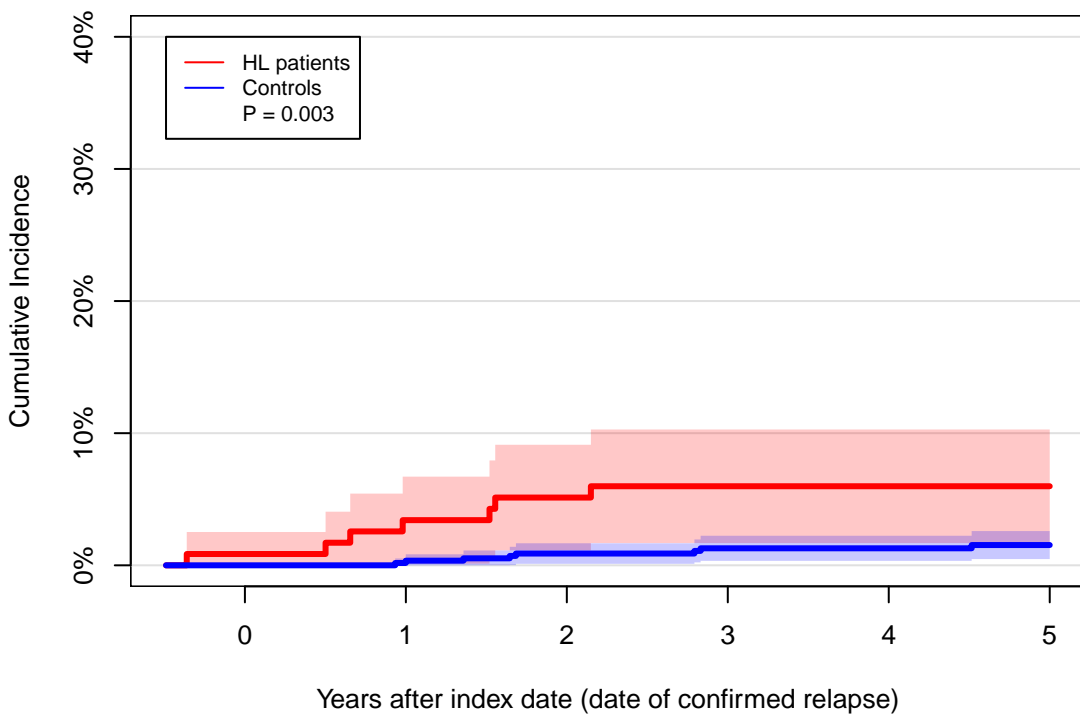

**Anxiolytics**

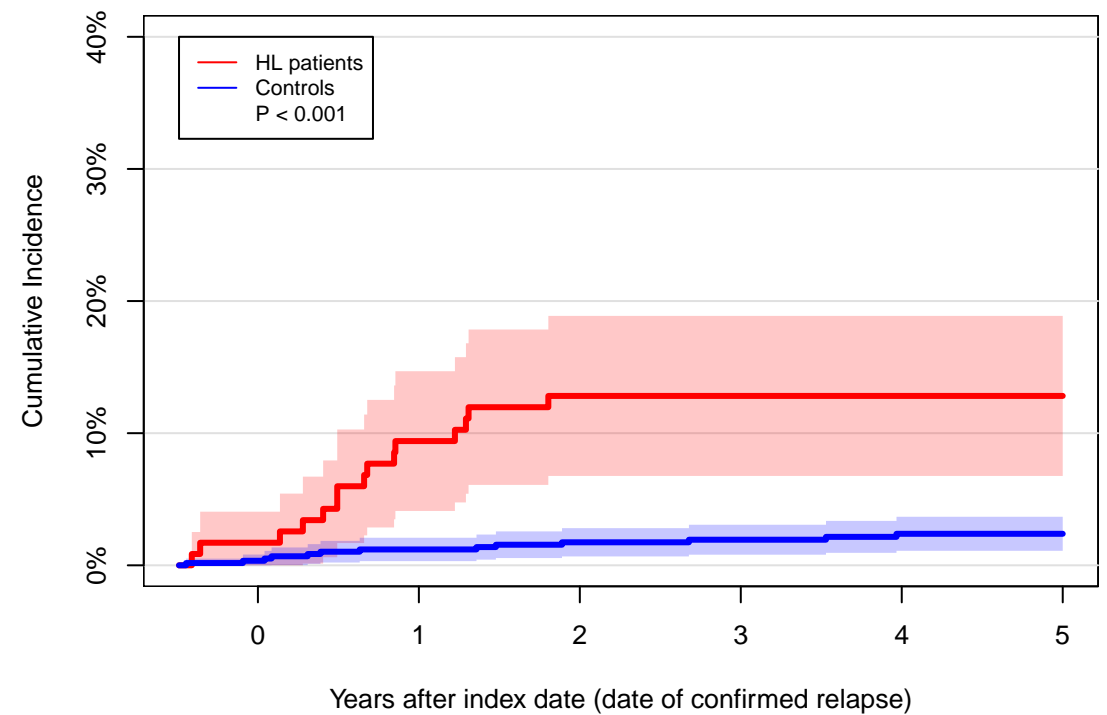

Supplement: Supplementary file 3 — Figure S3 [file CAM4-9-4395-s003.pdf]

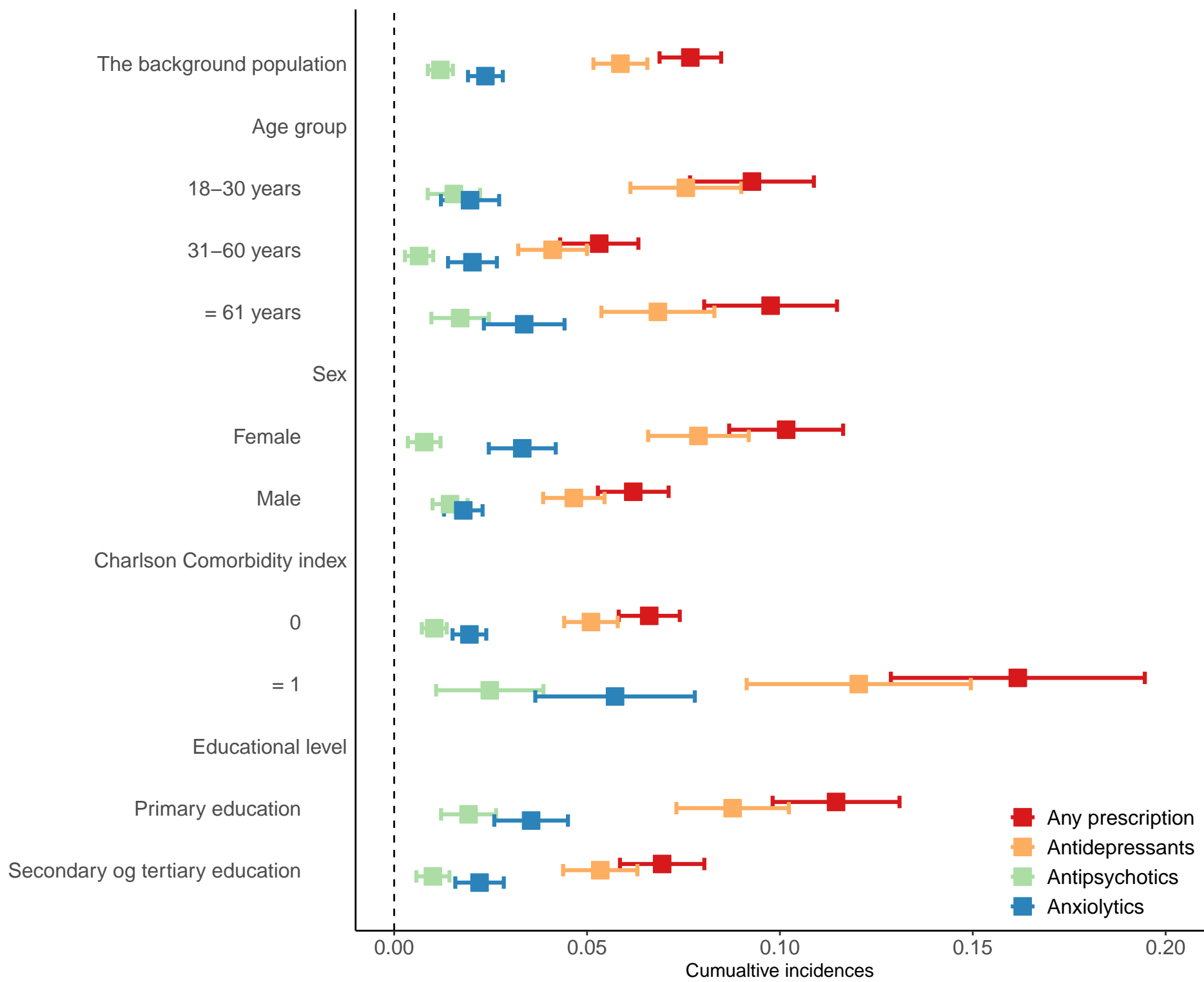

Supplement: Supplementary file 4 — Figure S4 [file CAM4-9-4395-s004.pdf]
